# Bacterial and fungal communities and contribution of physicochemical factors during cattle farm waste composting

**DOI:** 10.1002/mbo3.518

**Published:** 2017-07-24

**Authors:** Huhe, Chao Jiang, Yanpei Wu, Yunxiang Cheng

**Affiliations:** ^1^ Soil Fertilizer and Water‐Saving Institute Gansu Academy of Agricultural Sciences Lanzhou Gansu China; ^2^ The Ministry of Agriculture in Gansu Province Cultivated Land Conservation and Agricultural Environmental Science Observation Experiment Stations Wuwei Gansu China; ^3^ Institute of Grassland Research Chinese Academy of Agricultural Sciences Hohhot Inner Mongolia China; ^4^ State Key Laboratory of Grassland Agro‐ecosystems College of Pastoral Agriculture Science and Technology Lanzhou University Lanzhou Gansu China

**Keywords:** bacterial community, composting, fungal community, high‐throughput sequencing, physicochemical factors

## Abstract

During composting, the composition of microbial communities is subject to constant change owing to interactions with fluctuating physicochemical parameters. This study explored the changes in bacterial and fungal communities during cattle farm waste composting and aimed to identify and prioritize the contributing physicochemical factors. Microbial community compositions were determined by high‐throughput sequencing. While the predominant phyla in the bacterial and fungal communities were largely consistent during the composting, differences in relative abundances were observed. Bacterial and fungal community diversity and relative abundance varied significantly, and inversely, over time. Relationships between physicochemical factors and microbial community compositions were evaluated by redundancy analysis. The variation in bacterial community composition was significantly related to water‐soluble organic carbon (WSOC), and pile temperature and moisture (*p *<* *.05), while the largest portions of variation in fungal community composition were explained by pile temperature, WSOC, and C/N (*p *<* *.05). These findings indicated that those parameters are the most likely ones to influence, or be influenced by the bacterial and fungal communities. Variation partitioning analyses indicated that WSOC and pile temperature had predominant effects on bacterial and fungal community composition, respectively. Our findings will be useful for improving the quality of cattle farm waste composts.

## 1 INTRODUCTION

Composting technology, as an economical and effective treatment and resource utilization technology of organic solid waste, is one of the most important agriculture‐related research topics. Composting is the major process used for stabilizing agricultural and livestock wastes through the degradation of biodegradable components by microbial communities (Sun, Qian, Gu, Wang, & Gao, 2016; Tang, Shibata, Zhou, & Katayama, 2007; Tiquia, 2005; Yamamoto, Asano, Yoshii, Otawa, & Nakai, 2011). Generally, composting proceeds through three phases: (1) an initial phase in which the soluble, readily degradable compounds are rapidly broken down, causing a prompt increase in pile temperature from the moderate starting temperature (Nancy & Elaina, 1996); (2) a high‐temperature phase (active phase), in which the high temperatures accelerate the breakdown of proteins, fats, and complex carbohydrates such as cellulose and hemicellulose, the major structural molecules in plants (Lazcano, Gomez‐Brandon, & Dominguez, 2008); (3) a maturation phase in which the temperature decreases to the ambient temperature and that is characterized by a continued, slow degradation of organic compounds (Takaku, Kodaira, Kimoto, Nashimoto, & Takagi, 2006). The degradation of organic matter in a composting system occurs mainly through the interaction of different microorganisms. The composting reaction is a complex and variable biochemical process.

Through the action of the microorganisms, various environmental factors in the compost pile change over time. Among them, temperature is the most critical factor controlling the composting reaction rate because of its effect on the microbial metabolic rate and population structure (Palmisano & Barlaz, 1996). Certainly, other physicochemical factors also have substantive contributions. Numerous studies have assessed the impact of various physicochemical factors on microbial community composition in the composting process (Cahyani, Matsuya, Asakawa, & Kimura, 2003; Eiland, Klamer, Lind, Leth, & Bååth, 2001; Lei & VanderGheynst, 2000; Liang, Das, & McClendon, 2003; Tang et al., 2007; Zhang et al., 2011). Most of these studies suggested temperature and water‐soluble organic carbon (WSOC) to be the most influential (Cahyani et al., 2003; Ishii & Takii, 2003; Tang et al., 2007; Zhang et al., 2011). However, some studies have shown that the effect of moisture is higher than that of temperature (Liang et al., 2003). The initial pH of the compost substrate significantly affects the microbial community throughout the compost cycle (Lei & VanderGheynst, 2000). In addition, the initial C/N ratio of the substrate plays a role; higher fungal/bacterial ratios are observed in compost piles with high C/N ratios (Eiland et al., 2001). The effects of physicochemical factors on the bacterial and fungal communities are variable (Griffin, 1985; Herrmann & Shann, 1997; Klamer & Bååth, 1998), and may depend on the amount and type of nutrients available in the raw materials.

Although multiple studies have analyzed the changes in the microbial community during the composting cycle, these studies mainly employed phospholipid fatty acid (PLFA) profiling (Cahyani, Watanabe, Matsuya, Asakawa, & Kimura, 2002; Herrmann & Shann, 1997), denaturing gradient gel electrophoresis (DGGE) (Cahyani et al., 2003; Yamamoto et al., 2011), terminal restriction fragment length polymorphism (T‐RFLP) (Székely et al., 2009; Tiquia, 2005), and clone libraries (Li et al., 2013; Vivas, Moreno, Garcia‐Rodriguez, & Benitez, 2009). Although together, these techniques offer complementary approaches to directly or indirectly research microbial communities, they have a common drawback in that they detect only a small fraction of the microorganisms, thus limiting a comprehensive understanding of these communities (Li et al., 2013; McCaig, Glover, & Prosser, 2001; Nacke et al., 2011). Therefore, new and advanced methods are needed to investigate the differences in and dynamics of microbial community structures and compositions in composting.

In recent years, high‐throughput sequencing has been widely used to analyze the microbial communities in samples taken from various environments, such as soils (Sun, Xiao, Ning, Xiao, & Sun, 2015), wastewater treatment plants (Prevost et al., 2015), and composts (Holman, Hao, Topp, Yang, & Alexander, 2016). In comparison with traditional molecular biological techniques, high‐throughput sequencing allows to completely and accurately elucidate the microbial communities (Acosta‐Martínez, Dowd, Sun, Wester, & Allen, 2010).

To provide new insights for a better management of cattle manure composting, this study aimed to (1) investigate the bacterial and fungal community structures and compositions by high‐throughput sequencing of the 16S and 18S rRNA genes, and (2) analyze the effects of environmental factors as well as of key physicochemical factors on bacterial and fungal community changes during composting of cattle manure with edible residual of silage.

## 2 EXPERIMENTAL PROCEDURES

### 2.1 Composting process and sampling

The composting experiment was performed in June 2014. A field‐scale facility was provided by Yilanchun Cattle Industry Co. Ltd. (Dingxi, Gansu, China). Approximately 1,120 kg of cattle manure mixed with 210 kg of edible residual of corn straw silage to adjust the moisture content to 65% was used per compost pile, and three piles were included in the experiment. The characteristics of the raw materials are shown in Table 1. The composting material was piled and the composting process was monitored for 67 days. The material was stirred on the third after the start of composting for 4 consecutive days, once a week until day 38, and finally, on the 59th day. Compost samples were collected using a soil sampler on days 0, 8, 20, 30, 40, 50, and 67, at 30 cm from the surface; during these days (designated D1–D7), the pile temperature was 25, 72, 55, 34, 28, 27, and 20°C, respectively. On each sampling day, five samples were collected randomly and immediately pooled. Then, the samples were divided into two parts, placed on ice, and transferred to the laboratory. One part was stored at −80°C for microbial analysis by sequencing. The other part was used for the determination of physical and chemical properties of the compost.

**Table 1:** Characteristics of the raw composting materials

Resource materials	OC (g/kg)	TN (g/kg)	C/N ratio	pH	Moisture content (%)
Cattle manure	306.4	21.6	11.4	8.8	75.6
Edible residual of corn straw silage	465.1	8.4	55.4	nq	7.3

OC, total organic carbon; TN, total nitrogen; C/N, total organic carbon/total nitrogen; nq, sample not quantified.

### 2.2 Analysis of physicochemical parameters

The pile temperature was automatically measured hourly with a temperature and humidity data logger (RC‐4HC; Jingchuang Electronics Co., Ltd., Jiangsu, China) at 30 cm from the surface. The moisture content was determined as the wet weight minus the dry weight after oven drying at 105°C overnight. The dried samples were ground and inorganic carbon was removed using phosphoric acid. Total organic carbon (TOC) and total nitrogen (TN) were measured on an NC analyzer (Sumigraph NC‐900; Sumika Chemical Analysis Service, Tokyo, Japan) using the combustion method. Total carbon/nitrogen (C/N) was calculated from the values of TOC and TN. To measure the pH, 5 g of compost was suspended in 50 ml of distilled water and the pH of the supernatant was measured using a pH meter (AZ8685; AZ Instrument Corp., Taiwan). Ammonium and nitrate were extracted with 2 mol/L KCl and determined on an automated flow analysis instrument (FIAstar 5000 Analyzer; Foss Tecator, Hillerød, Denmark). WSOC was determined by the method described by Zhang et al. (2011). Available potassium (A‐K) and available phosphorus (A‐P) were extracted with 1 mol/L HNO_3_ (Pratt, 1965) and a 2% (m/V) citric acid solution (Drouillion & Merckx, 2003) and determined by flame photometry (model 410 photometer; Corning, Halstead, Essex, England) and colorimetry, respectively.

### 2.3 DNA collection and high‐throughput sequencing

Genomic DNA was extracted using the PowerSoil DNA Isolation Kit (Mo Bio Laboratories, Solana Beach, CA, USA) per the manufacturer's instructions. Each compost sample was extracted in triplicate; the extracts were mixed into a single DNA sample and detected by 1% agarose gel electrophoresis. Bacterial 16S rRNA and fungal 18S rRNA genes were amplified using the primers 338F‐806R (Huws, Edwards, Kim, & Scollan, 2007) and 817F‐1196R (Rousk et al., 2010), respectively. PCR was carried out on a GeneAmp 9700 PCR system (Applied Biosystems, Foster City, CA, USA). Each sample was amplified in triplicate, and the amplified products were mixed and detected by 2% agarose gel electrophoresis. DNA was purified from the gel by using the AxyPrep DNA gel extraction kit (Axygen Biosciences, Union City, CA, USA), washed with Tris‐HCl, and verified by 2% agarose gel electrophoresis. Sequencing was conducted by Shanghai Majorbio Bio‐pharm Technology (Shanghai, China), using an Illumina MiSeq platform (San Diego, CA, USA).

### 2.4 Data analysis

Sequences that were shorter than 200 bp, had ambiguous bases, or had an average mass <25 were removed using the mothur software (Schloss et al., 2009). Chimeric sequences were removed using USEARCH v7.1 (Edgar, 2010). Operational taxonomic units (OTUs) were defined by clustering, with the threshold set at 97% identity, and diversity (Shannon–Wiener and Simpson's diversity Index) and relative abundance (Chao1 and ACE) were estimated using the Qiime software (Caporaso et al., 2010). OTUs were classified using the SILVA database containing bacterial and fungal ribosomal RNA sequences (version 119) (Pruesse et al., 2007). High‐throughput sequencing data have been deposited in the NCBI Sequence Read Archive (BioProject ID PRJNA353324, study accession number SRP093410).

### 2.5 Statistical analysis

The Canoco program for Windows 4.5 (Biometris, Wageningen, the Netherlands) was used for redundancy analysis (RDA) and variation partitioning analysis. First, detrended correspondence analysis was carried out to decide between a linear or unimodal response model for the microbial sequencing data. The length of the gradient was 3.89 for bacterial and 3.19 for fungal species data, respectively, which indicated that the two models are suitable. The linear response model was selected in this study. RDA was performed with default settings. Monte Carlo reduced model tests with 499 unrestricted permutations were used to statistically evaluate the significance levels. Variation partitioning was performed to discriminate the influence of each significant factor determined by partial RDA; each significant physicochemical factor was separately used as constraining variable, while other significant variables were used as covariables. The analysis was repeated with inverted constraining variable and covariables to estimate significant physicochemical factors individually and collectively. Regression analysis was performed using SPSS (version 19.0; SPSS, Chicago, IL, USA). Linear discriminant analysis (LDA) coupled with effect size measurements (LEfSe) analysis was conducted to search for statistically different biomarkers between groups (Segata et al., 2011). *p *<* *.05 was regarded significant for all analyses.

## 3 RESULTS

### 3.1 Physicochemical characteristics

**Figure 1:**
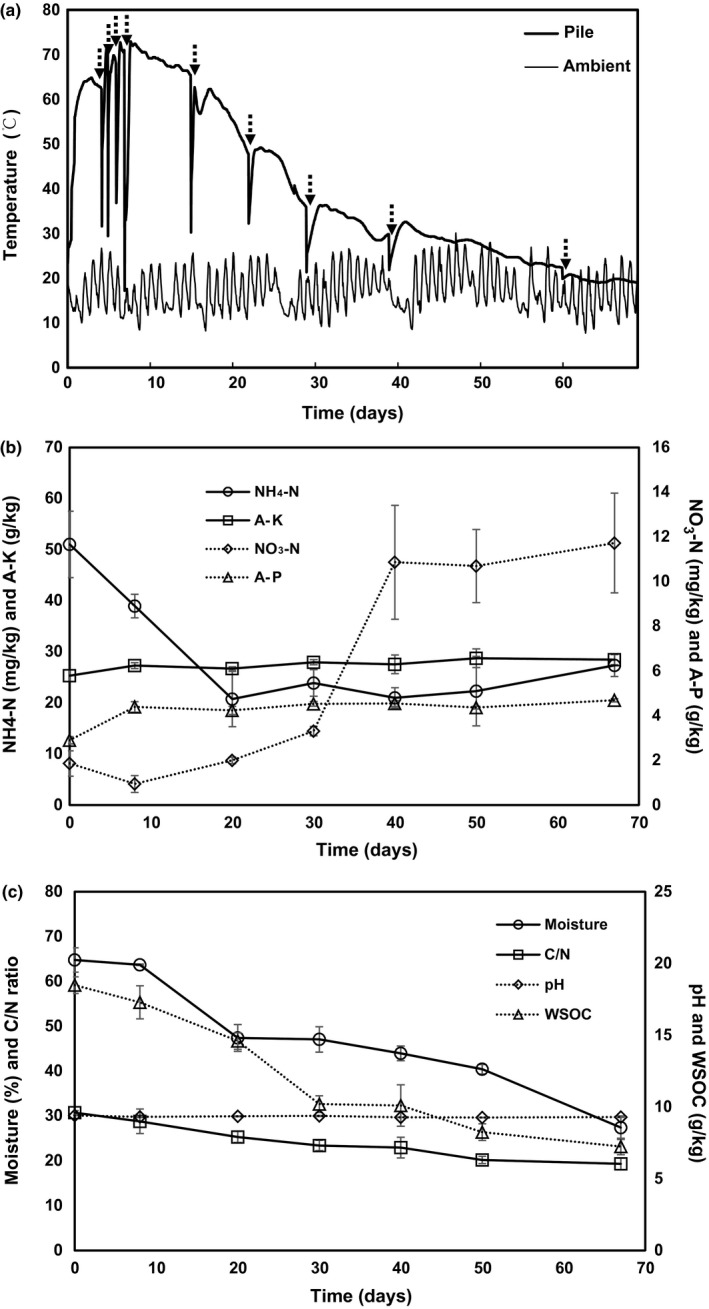
Dynamics of physicochemical factors throughout the composting process. (a) Pile and ambient temperature—arrows indicate each time of compost turn over—, (b) NH_4_‐N, NO_3_‐N, A‐K, and A‐P, (c) moisture, C/N ratio, pH, and water‐soluble organic carbon

The temperature dynamics during the composting process are shown in Figure 1a. The pile temperature gradually increased to 65°C during the first 3 days. The peak temperature during the entire composting period was 72°C. High temperatures (>55°C) were maintained for approximately 20 days, which met the hygiene requirements for organic waste (USEPA, 1994). The compost temperature gradually decreased after approximately 60 days to close to the ambient temperature, after which the pile temperature remained stable. Along with the rapid increase in pile temperature, the NH_4_‐N content gradually decreased from an initial 51.0 to 20.8 mg/kg on day 22, after which no obvious changes were observed. In contrast, nitrate did not obviously change during the high‐temperature period; however, when the temperature dropped to about 40°C, it significantly increased from 3.31 to 10.9 mg/kg (Figure 1b). With the decrease in temperature, pile moisture, WSOC, and C/N ratio decreased from 64.8% to 27.4%, from 18.5 to 6.25 g/kg, and from 30.7 to final 17.3, respectively (Figure 1c). A‐P, A‐K, and pH showed no obvious changes during the composting process (Figure 1b).

### 3.2 Dynamic changes in microbial community abundance and diversity

DNA was extracted from compost samples taken at different temperature stages of the composting process. The MiSeq platform was used for 16S/18S rRNA gene sequencing. We obtained 417,357 and 356,386 quality‐filtered and chimera‐checked 16S/18S rRNA gene sequences with an average length of 440 and 400 bp across all samples, respectively. The number of 16S rRNA sequences per sample varied from 10,042 to 24,346, and the number of 18S rRNA sequences per sample varied from 12,101 to 29,553. In total, 471 bacterial OTUs and 91 fungal OTUs were obtained from the 21 DNA samples.

**Figure 2:**
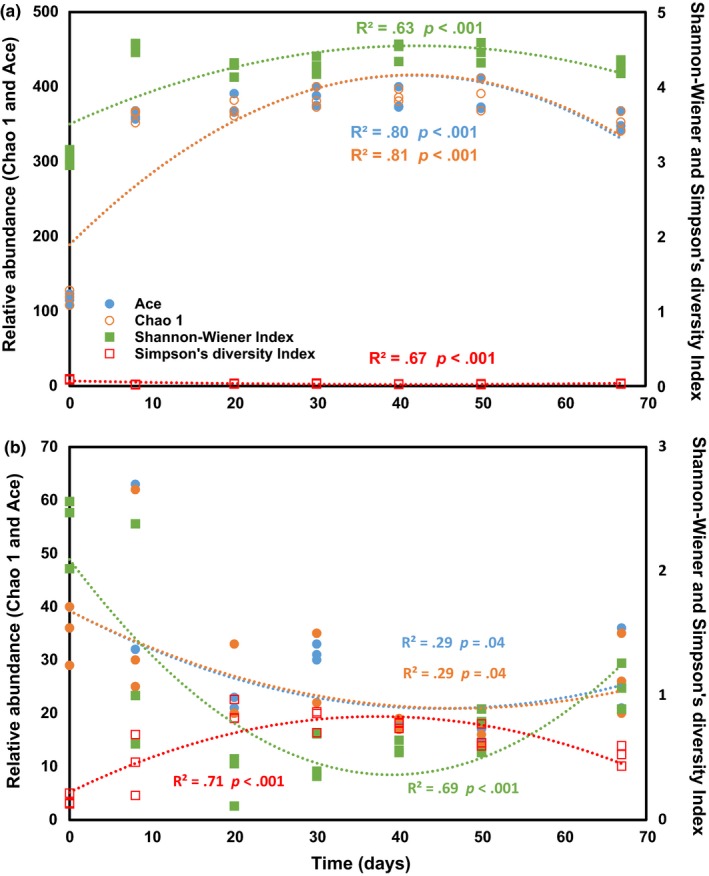
Changes in relative abundance and diversity of bacterial (a) and fungal communities (b) during composting

Bacterial and fungal community relative abundance (Ace and Chao1) and diversity (Shannon and Simpson) index values revealed dynamic changes at different temperature stages (Figure 2). The relative abundance and diversity of bacteria as well as fungi significantly changed parabolically with composting time. The relative abundance and diversity of the bacterial community first increased and then decreased with time (Figure 2a), while changes in the fungal community were in the opposite direction (Figure 2b).

### 3.3 Phylum‐level taxonomic distribution

**Figure 3:**
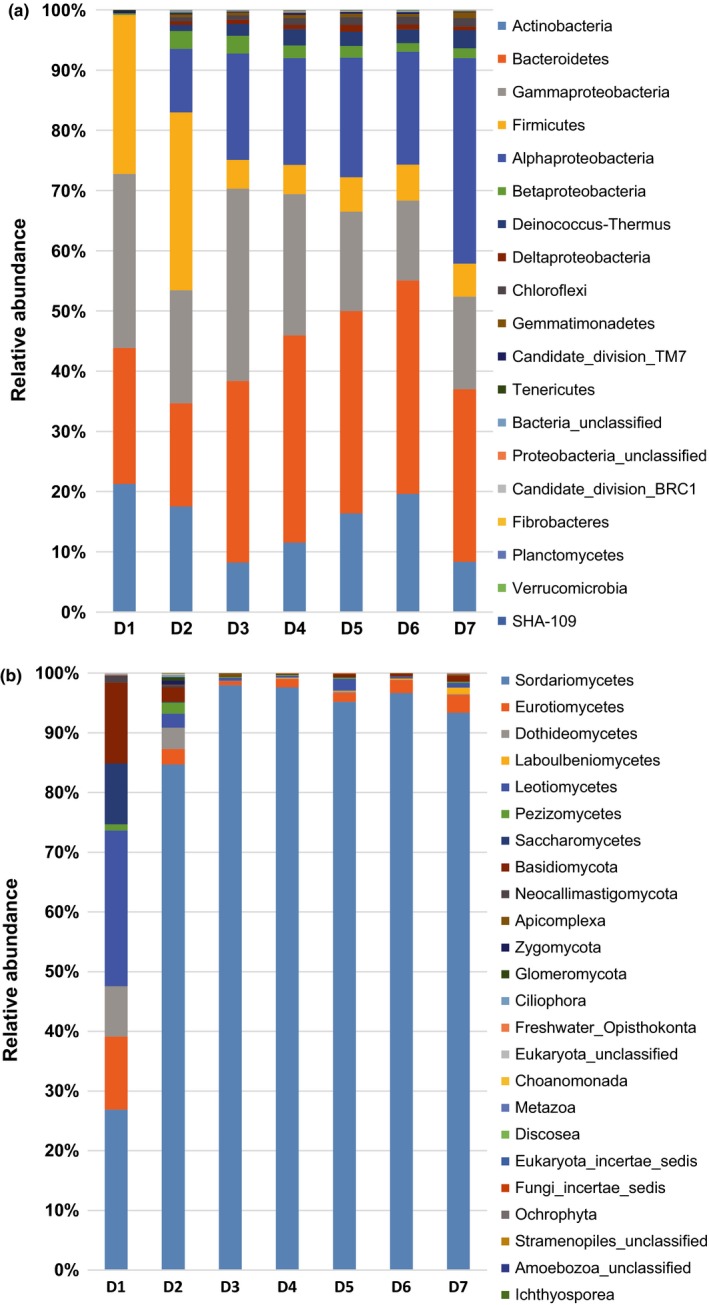
Relative abundance of the dominant bacterial (a) and fungal (b) phyla in the composting samples

*Proteobacteria* and *Ascomycota* were the main classes considered in the phylum‐level analysis because they represented 30%–53% and 84%–99% of all phyla in the compost samples (Figure 3). This narrowing down phylogenetically allows providing more detailed information. The compost samples harbored diverse lineages of bacterial and fungal phyla; we identified 19 bacterial phyla (*Proteobacterial* classes) and 24 fungal phyla (*Ascomycota* classes). The predominant phyla in the bacterial and fungal communities were largely consistent during the composting. However, differences in relative abundances were observed (Figure 3a,b). The 10 most abundant groups of bacteria were *Actinobacteria*,* Bacteroidetes*,* Gammaproteobacteria*,* Firmicutes*,* Alphaproteobacteria*,* Betaproteobacteria*,* Deinococcus–Thermus*,* Deltaproteobacteria*,* Chloroflexi*, and *Gemmatimonadetes*, and the most abundant fungal taxa were *Sordariomycetes*,* Leotiomycetes*,* Basidiomycota*,* Saccharomycetes*,* Eurotiomycetes*,* Pezizomycetes*,* Laboulbeniomycetes*,* Apicomplexa*,* Neocallimastigomycota*, and unclassified *Eukaryota*. In particular, the class *Sordariomycetes* accounted for about 27% in the initial sample, while it accounted for more than 85% in samples taken at later time points (Figure 3b).

#### 3.3.1 Composting phase‐dependent enrichments in bacterial and fungal communities

Apart from determining α‐ and β‐diversities, another major goal of comparing microbial communities is to determine the professional community/communities in samples. For this purpose, we used LEfSe. This tool allows analyzing microbial community data at any clade; however, as analysis of the large number of OTUs detected in this study would be computationally too complex, statistical analysis was performed only from the domain to the genus level.

**Figure 4:**
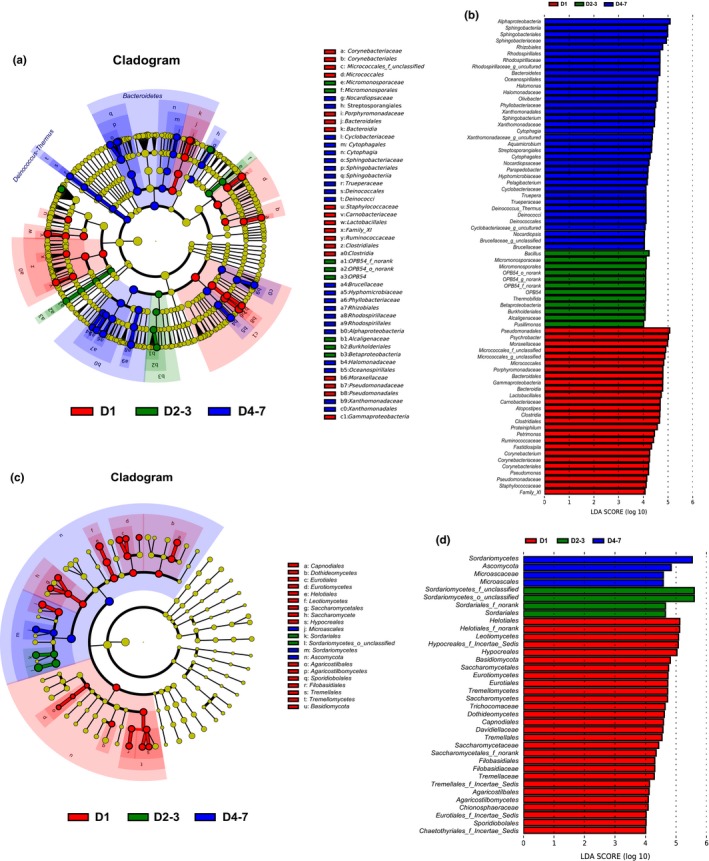
Cladogram showing the phylogenetic distribution of the bacterial and fungal lineages associated with compost in the three different stages of composting (a and c). Indicator bacteria with linear discriminant analysis scores of ≥4 in bacterial and fungal communities associated with compost in the three different stages of composting (b and d). Different‐colored regions represent different stages (red, D1; green, D2–3; blue, D4–7). Circles indicate phylogenetic levels from domain to genus. The diameter of each circle is proportional to the abundance of the group

The composting process comprised different temperature stages. Therefore, we divided the data into three groups—initial (D1), thermophilic (D2–3), and maturation phase (D4–7)—that were analyzed using the LEfSe tool. Groups were shown in cladograms, and LDA scores of ≥4 were confirmed by LEfSe (Figure 4). In the three stages of composting, significantly enriched bacterial and fungal taxa varied. Additionally, we used primers to detect other eukaryotic groups, including *Stramenopiles*,* Amoebozoa*,* Holozoa*,* Alveolata*, and *Opisthokonta*; however, no significant differences in these groups were found among the three phases of composting (Figure 4c).

### 3.4 Redundancy and variation partitioning analyses

**Figure 5:**
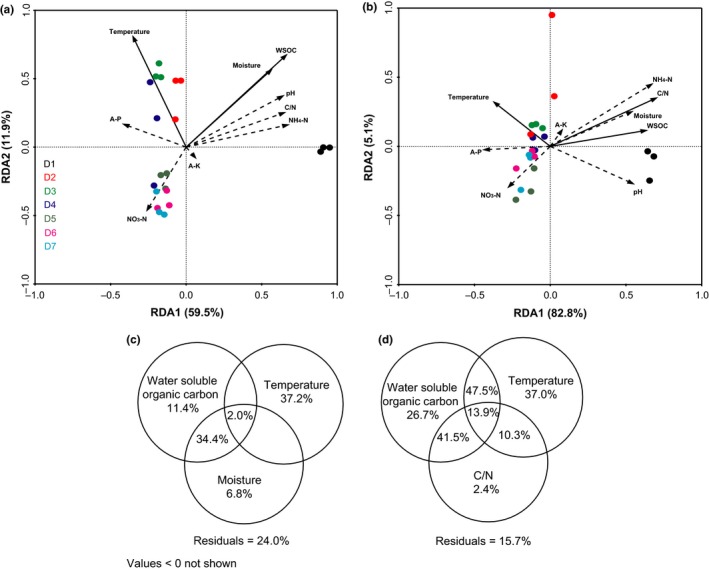
Redundancy analysis of MiSeq data (symbols) and environmental characteristics (arrows). Bacterial and fungal communities are shown in a and b, respectively. The values of axes 1 and 2 are the percentages explained by the corresponding axis. Analysis of the level of contribution of significant factors (solid arrows) to changes in bacterial (c) and fungal (d) communities

To determine to what extent physicochemical factors affect microbial community composition during composting, both bacterial and fungal OTU‐level structures were analyzed by redundancy analysis. Bacterial samples were more obviously clustered according to the composting stage than the fungal samples (Figure 5a,b). The first axis explained 59.5% and 82.8% of the bacterial and fungal diversity observed, while the second axis explained 11.9% and 5.1% of the variation. The eight analyzed environmental variables were included in the RDA biplot. Temperature and WSOC had significant effects on the bacterial and fungal community changes, while moisture and C/N significantly affected the bacterial and fungal community compositions, respectively. Individual and collective contributions of factors determined by RDA to significantly contribute to bacterial and fungal community changes were analyzed by variation partitioning. The significant factors obtained from the RDA model statistically explained up to 76.0% and 84.3% of the variation in the bacterial (temperature, WSOC, and moisture) and fungal (temperature, WSOC, and C/N) communities, respectively. Figure 5c and d show the percentages of variation explained by each of these parameters individually and by combined actions. Pile temperature solely explained 37.2% (*p *=* *.002), WSOC 11.4% (*p *=* *.002), and moisture 6.8% (*p *=* *.002) of the variation in bacterial community. The variation shared by temperature, WSOC, and moisture was 2.0%, and that shared by WSOC and moisture was 34.4%. Pile temperature solely explained 37.0% (*p *=* *.008) of the variation in fungal community composition, while WSOC and C/N explained 26.7% (*p *=* *.002) and 2.4% (*p *=* *.004), respectively. The variation shared by WSOC and C/N, and temperature and C/N was 41.5% and 10.3%, respectively. No variation was shared by temperature, WSOC and C/N, and by temperature and WSOC in the fungal data.

## 4 DISCUSSION

This study used high‐throughput sequencing to dissect bacterial and fungal community changes and the relationships with the physicochemical factors driving these changes during cattle manure composting. Composting processes generally have different temperature phases. The initial increase in temperature is conducive to some, but not all, microbes present in the pile at this stage. Karadag et al. (2013) found that the temperature gradient significantly affects the microbial population during municipal solid waste composting. Steger, Jarvis, Vasara, Romantschuk, and Sundh (2007) and Shukla, Rai, and Dubey (2009) observed that during the high‐temperature stage, microbial biomass and diversity are reduced owing to a high‐temperature screening effect. In addition, Tiquia (2005) found that bacterial and fungal communities respond differently to the high temperatures in this phase in a mixed composting system of cattle and horse manure. Accordingly, this study showed a significant screening effect of high temperatures on the fungal community. The bacterial community showed an opposite response: relative abundance and diversity were increased at high temperatures. The increase in pile temperature is mainly attributable to heat dissipation by the bacteria, which are able to grow rapidly on the soluble proteins and other readily available nutrients (Epstein, 1997; Golueke,^1992^). In other words, the nutritional conditions in the cattle manure composting system seem to sustain bacterial growth so as to overcome the screening effect of high temperatures on the bacteria.

With the changes in environmental factors during the transition to the high‐temperature stage, the originally dominant microbial dominant taxa associated with the compost materials were gradually replaced. In this study, the most abundant bacteria in the thermophilic stage were *Firmicutes* (*Bacillus* and OPB54). An increase in low‐G+C gram‐positive bacteria from *Firmicutes* is often found in this stage, even in different composting systems utilizing various organic wastes (de Gannes, Eudoxie, & Hickey, 2013; Takaku et al., 2006). Other studies have also suggested that the genus *Bacillus* is the most dominant bacterial taxon in not only the thermophilic phase but throughout the composting process (Godden, Ball, Helxenstein, McCarthy, & Penninckx, 1992; Juteau, Tremblay, Villemur, Bisaillon, & Beaudet, 2004). In this study, the A‐P content at the initial stage was significantly lower than that of the other composting stages, the same result was obtained in the study of vermi composting and aerobic composting, and the phosphorus solubilizing bacteria *Bacillus cereus* IP4 strain was isolated (Hussain et al., 2016). *Actinobacteria* (genus *Thermobifida* and order *Micromonosporales* and its family *Micromonosporaceae*) were the second most predominant bacteria at the thermophilic stage. These bacteria can form spores, which allow them to tolerate high temperatures (Tian et al., 2013).

The phylum *Proteobacteria* was the most dominant throughout the composting process, with various classes being enriched in different stages; *Gammaproteobacteria* in the initial stage, *Betaproteobacteria* in the thermophilic phase, and *Alphaproteobacteria* during maturation. Of *Betaproteobacteria*, only the genus *Pusillimonas* was significantly enriched in the high‐temperature stage. Similarly, Lv, Xing, Yang, and Zhang (2015) observed a higher abundance of *Pusillimonas* in the high temperature than in the mature stage. Although the class *Gammaproteobacteria* and its order *Pseudomonadales* were significantly more abundant in the raw materials than in other composting stages, in the cooling stage, succession of the orders *Oceanospirillales* and *Xanthomonadales* was observed. This finding confirmed that some mesophilic bacteria could enter dormancy during the thermophilic period and resume growth during the cooling phase (Yamada et al., 2008). The abundance of *Alphaproteobacteria* (genera uncultured *Rhodospirillaceae*, unclassified *Brucellaceae*,* Pelagibacterium*, and *Aquamicrobium*) was also significantly higher in the mature than in the thermophilic phase. Danon, Franke‐whittle, Insam, Chen, and Hadar (2008) reported that *Alphaproteobacteria* groups were high in numbers in composts from all curing stages. The genera *Olivibacter*,* Sphingobacterium*,* Parapedobacter*, uncultured *Cyclobacteriaceae*, and *Nocardiopsis* of *Bacteroidetes*, and the genus *Truepera* of *Deinococcus–Thermus* were significantly enriched in the maturation stage. Previous studies have revealed that these bacteria can decompose high‐molecular‐weight organic matter such as starch, cellulose, proteins, xylan, and chitin (Eichorst et al., 2013, 2014; Takaku et al., 2006).

As for the fungi, unclassified *Sordariomycetes* and *Microascaceae* of *Sordariomycetes* were enriched in the high‐temperature and maturation stages, respectively. The class *Sordariomycetes* of *Ascomycota* dominated the composting process, accounting for 26.8%, 91.2%, and 95.8% in the raw material, high‐temperature stage, and maturation, respectively. de Gannes et al. (2013) also observed that *Sordariomycetes* was the predominant class in different phases of composting of various materials. Many members of *Sordariomycetes* can break down lignin and cellulose (Zhang et al., 2006) and thus are involved in wood decomposition (Santiago‐Rodriguez, Toranzos, Bayman, Massey, & Cano, 2013).

The bacterial community was distinctly separated from the fungal community in overall abundance during the three stages of composting, indicating it was likely more sensitive to temperature fluctuations than the fungal community. Not only microbial growth is dependent on the temperature; the community structure within composts is also controlled by various environmental factors. As expected, RDA showed that the composition of the microbial population during composting is mainly controlled by temperature. In addition, WSOC was detected to significantly correlate with the distributions of both bacterial and fungal communities. WSOC has been reported as an important factor affecting microbial community structure and metabolic type (Ishii & Takii, 2003; Maeda, Morioka, & Osada, 2009). Finally, moisture had a significant effect on bacterial community composition in this study. It is known that water is the medium for nutrient transportation and metabolic reactions in microorganisms. Nutrient availability is affected by the water content in the microenvironment of the microbes, especially in the thin liquid layers on particle surfaces (Ghaly, Dave, & Zhang, 2012). However, no significant correlation between moisture and fungal community composition was obtained in this research, indicating that bacterial communities may be more sensitive to fluctuations in moisture content than fungal communities. The C/N ratio is another factor essential to the composting process and is an indicator of compost quality. The microbial community structure was affected by the initial C/N ratio, with higher substrate C/N benefiting fungal growth (Bossuyt et al., 2001; Eiland et al., 2001; Six, Frey, Thiet, & Batten, 2006). In this study, fungal abundance decreased with the reduction in C/N ratio during the composting process. In addition, WSOC had a strong interaction with moisture (34.4%), and C/N (41.4%), suggesting interrelatedness of their effects; a change in moisture affects the dissolved amount of WSOC, which in turn influences C/N. Changes in available carbon and volatilization of ammonia during the composting process were influenced by temperature and C/N, indicating an interaction between C/N and temperature.

In conclusion, high‐throughput 16S/18S rRNA sequencing indicated that the primary phyla in compost samples of different temperature stages included *Proteobacteria*,* Bacteroidetes*,* Firmicutes*, and *Actinobacteria* of bacteria, and *Ascomycota* of fungi. Bacterial and fungal diversity and relative abundance significantly changed parabolically with the proceeding of composting. Various physicochemical parameters also varied to different degrees with composting progress. Both pile temperature and WSOC showed a predominant effect on bacterial and fungal community composition. Moisture and C/N significantly contributed to the distribution of bacterial and fungal communities, respectively. Furthermore, WSOC had a strong interaction with moisture and C/N. Thus, the rational management of these physicochemical factors may contribute to maturation rate and quality improvement in cattle farm waste composting.

## CONFLICT OF INTEREST

There are no conflicts of interest.
