# Supplementary material for: Bacterial and fungal communities and contribution of physicochemical factors during cattle farm waste composting
Source: Microbiologyopen. 2017 Jul 24;6(6):e00518. doi: 10.1002/mbo3.518 (PMC5727367; doi:10.1002/mbo3.518)
Supplement: Supplementary file 1 [file MBO3-6-na-s001.docx]

**Bacterial and fungal communities and contribution of physicochemical factors during cattle farm waste composting**

Huhe^1,2^, Chao Jiang^3^, Yanpei Wu^4^, Yunxiang Cheng^4^ ^*^

^1^ Soil Fertilizer and Water-Saving Institute, Gansu Academy of Agricultural Sciences, Lanzhou, Gansu, 730070, China

^2^ The Ministry of Agriculture in Gansu Province Cultivated Land Conservation and Agricultural Environmental Science Observation Experiment Stations, Wuwei, Gansu, 733017, China

^3^ Institute of Grassland Research, Chinese Academy of Agricultural Sciences, Hohhot, Inner Mongolia, 010010, China

^4^ State Key Laboratory of Grassland Agro-ecosystems, College of Pastoral Agriculture Science and Technology, Lanzhou University, Lanzhou,

Gansu, 730020, China

^*^For correspondence: E-mail chengyx@lzu.edu.cn; Tel. (+86) 931 8912925; Fax (+86) 931 8910979

**Supplementary Table 1**: Relative abundance and diversity of bacterial and fungal communities

| Sampling time (day) | Bacterial* | | | |  | Fungal* | | | |
| --- | --- | --- | --- | --- | --- | --- | --- | --- | --- |
|  | Ace | Chao 1 | Shannon-Wiener diversity Index | Simpson's diversity Index |  | Ace | Chao 1 | Shannon-Wiener diversity Index | Simpson's diversity Index |
| 0 | 115.33±7.51 | 118.33±10.02 | 3.05±0.11 | 0.09±0.01 |  | 35.00±5.57 | 35.00±5.57 | 2.35±0.29 | 0.16±0.05 |
| 14 | 361.67±5.69 | 361.33±8.14 | 4.54±0.07 | 0.02±0.00 |  | 29.00±3.61 | 28.33±2.89 | 1.33±0.93 | 0.45±0.25 |
| 22 | 375.00±13.89 | 370.00±10.82 | 4.25±0.10 | 0.03±0.01 |  | 21.33±1.53 | 24.33±7.51 | 0.35±0.21 | 0.87±0.09 |
| 29 | 387.67±12.50 | 384.67±13.87 | 4.28±0.12 | 0.03±0.01 |  | 31.33±1.53 | 30.67±7.51 | 0.47±0.19 | 0.81±0.09 |
| 38 | 382.33±15.31 | 388.00±8.19 | 4.48±0.13 | 0.02±0.00 |  | 17.67±0.58 | 17.67±1.15 | 0.58±0.05 | 0.76±0.03 |
| 47 | 385.33±23.12 | 390.00±21.52 | 4.45±0.14 | 0.02±0.01 |  | 16.67±1.53 | 16.00±2.00 | 0.74±0.18 | 0.66±0.10 |
| 67 | 352.67±13.61 | 353.67±13.01 | 4.26±0.09 | 0.03±0.00 |  | 27.67±7.64 | 27.00±7.55 | 1.07±0.19 | 0.52±0.08 |

*The values shown for management factors are mean ± standard errors.
